# MicroRNA-429 Modulates Hepatocellular Carcinoma Prognosis and Tumorigenesis

**DOI:** 10.1155/2013/804128

**Published:** 2013-09-24

**Authors:** Xiao-Ying Huang, Jin-Guang Yao, Hong-Dong Huang, Chao Wang, Yun Ma, Qiang Xia, Xi-Dai Long

**Affiliations:** ^1^Department of Pathology, Youjiang Medical College for Nationalities, Baise 533000, China; ^2^Division of Nephrology, Beijing Shijitan Hospital, Capital Medical University, Beijing 100038, China; ^3^Department of Medicine, The Affiliated Hospital of Youjiang Medical College for Nationalities, Baise 533000, China; ^4^Department of Pathology, The First Affiliated Hospital, Guangxi Medical University, Nanning 530021, China; ^5^Department of Liver Surgery, Renji Hospital, School of Medicine, Shanghai Jiao Tong University, Shanghai 200127, China

## Abstract

MicroRNA-429 (miR-429) may modify the development and progression of cancers; however, the role of this microRNA in the hepatocellular carcinoma (HCC) has not been well elaborated. Here, we tested miR-429 expression in 138 pathology-diagnosed HCC cases and SMMC-7721 cells. We found that miR-429 was upregulated in HCC tumor tissues and that the high expression of miR-429 was significantly correlated with larger tumor size (odd ratio (OR), 2.70; 95% confidence interval (CI), 1.28–5.56) and higher aflatoxin B1-DNA adducts (OR = 3.13, 95% CI = 1.47–6.67). Furthermore, this microRNA overexpression modified the recurrence-free survival and overall survival of HCC patients. Functionally, miR-429 overexpression progressed tumor cells proliferation and inhibited cell apoptosis. These results indicate for the first time that miR-429 may modify HCC prognosis and tumorigenesis and may be a potential tumor therapeutic target.

## 1. Introduction

Primary liver cancer is the sixth most commonly occurring cancer worldwide, with an estimated 600,000 new cases per year [1–3]. Because of the very poor prognosis and the same number of deaths, this tumor is the third most common cause of cancer deaths in the world [2, 3]. Liver cancer is histopathologically classified into two major types, hepatocellular carcinoma (HCC) and cholangiocellular carcinoma. HCC often exhibits blood metastasis and recurrence [4–6]. Therefore, improvement in the therapy of recurrent or metastatic HCC now depends on improving our understanding of the complex molecular mechanisms governing the progression and aggressiveness of the disease.

Over the last several decades, it has been recognized that multiple risks, including hepatitis B virus (HBV) and hepatitis C virus (HCV) infection, chemical carcinogen aflatoxin B1 (AFB1), and genetic abnormalities, are implicated in the multistep process of liver carcinogenesis [5–7]. Increasing evidence has shown that microRNA may play an important role in the tumorigenesis of this malignant [8–12], while microRNA-429 (miR-429), a member of the microRNA-200 family of microRNAs, can hinder the expression of transcriptional repressors SIP1/ZEB213 and ZEB1/deltaEF1 and regulate epithelial-mesenchymal transition [13, 14]. Recent studies have shown that downregulation of miR-429 may be an important late step in tumour progression [14]. Increasing data exhibit that dysregulation of this microRNA expression can modify tumor prognosis possibly through regulating cell proliferation and apoptosis [13, 15–18]. However, association between this microRNA and HCC has not yet been elucidated. Here, we evaluated whether miR-429 expression modified HCC clinicopathological features and prognosis and explored the effects of this microRNA on cancer cell proliferation and apoptosis.

## 2. Materials and Methods

### 2.1. Patient and Followup

This study was approved by the ethics committees of the hospitals involved in this study. All activities involving human subjects were carried out under full compliance with government policies and the Helsinki Declaration. A total of 138 HCC patients, including 65 patients previously studied [19, 20], were included in the present study. All cases were identified through hepatosurgery, hepatopathology, oncology, hepatology centers, and through cancer registries in the affiliated hospitals of the two main medical colleges in Guangxi (namely, Guangxi Medical University and Youjiang Medical College for Nationalities) between January 2004 and December 2005. All of the cases were confirmed by histopathological diagnosis in 100% of the HCC cases with the I-II tumor-nodes-metastasis (TNM) stage and had undergone the same curative resection treatment, according to Chinese Manage Criteria of HCC [21]. After giving written consent, demographic information (including sex, age, ethnicity, and HBV and HCV infection) and clinical pathological data (including cirrhosis, tumor size, and tumor stage) were collected in the hospitals using a standard interviewer-administered questionnaire and/or medical records [19, 20, 22]. Surgically removed tumor samples and adjacent noncancerous tissue samples (at least 5 cm from the margin of the tumor) of all cases were collected for analyzing miR-429 expression levels and AFB1-DNA adduct levels. In this study, those hepatitis B surface antigens (HBsAg) positive and anti-HCV positive in their peripheral serum were defined as groups infected with HBV and HCV. Liver cirrhosis was diagnosed by pathological examination, and stages of tumor were confirmed according to the TNM staging system.

For survival analysis, all patients were followed and underwent serial monitoring every 2 months for the first 2 years and semiannually thereafter for detection of any recurrence. The last follow-up day was set on August 31, 2012, and survival status was confirmed by means of clinic records and patient or family contact. In this study, the duration of recurrence-free survival (RFS) was defined as from the date of curative treatment to the date of tumor recurrence or last known date alive, whereas the duration of overall survival (OS) was defined as from the date of curative treatment to the date of death or last known date alive. More detailed information was described in our previous studies [19, 20].

### 2.2. AFB1-DNA Adducts Analysis

Genomic DNA was extracted from HCC tumor tissues and SMMC-7721 cells in a 1.5 mL microcentrifuge tube for deparaffinization and proteinase K digestion, as described by standard procedures (Protocol #BS474, Bio Basic, Inc., Ontario, Canada). DNA was treated according to previously reported procedure, and N-7 adduct was converted to AFB1-FAPy adduct. AFB1-FAPy adduct was quantitated by competitive enzyme-linked immunosorbent assay (ELISA) using monoclonal antibody 6A10 (Novus Biologicals LLC, catalog # NB600-443) [23]. For analysis, AFB1-DNA adduct levels were divided into two groups: low level (≤3.00 *μ*mol/mol DNA) and high level (≥3.01 *μ*mol/mol DNA), according to the average value of AFB1-DNA adduct levels among cases.

### 2.3. MiR-429 Expression Assay

Quantitative reverse transcription-PCR was used to test miR-429 expression. Total RNA was isolated from tissue or cell cultures, using PureLink RNA Mini Kit (cat# 12183018A, Ambion, USA) according to manufacturer's instructions. For mRNA quantitation, RNA was reversed transcribed into cDNA using High Capacity cDNA Reverse Transcription Kit (cat# 4368814, Invitrogen Grand Island, NY, USA) and TaqMan MicroRNA Reverse Transcription Kit (with including miR-429 primer and U6 primer, cat# 4366596, Applied Biosystems (ABI), Carlsbad, CA) according to the manufacturer's instructions. Real-time quantitative PCR analysis was performed using standard protocols on a Bio-Rad iCycler iQ5 Detection System. Mature miR-429 expression was assessed using TaqMan microRNA assays (cat# 4427975, ABI) with human U6 as the endogenous control. PCR reactions were run in a 5 mL final volume containing 1 × TaqMan Universal Master Mix II (cat# 4440041, ABI), 1 × TaqMan microRNA probes and primers, and about 15 ng of cDNA. Cycling conditions were 95°C for 30 s and 50 cycles of 95°C for 15 s and 60°C for 1 min. All reactions were conducted in triplicate, and controls were performed with no template or no reverse transcription for each gene. The cycle number at which the reaction crossed an arbitrarily placed threshold (CT) was determined for each gene. For tissue samples, the relative amount of miR-429 to U6 was calculated as 2^−ΔCt^ method, where ΔCt = (Ct_miR-429_ − Ct_U6_). For analysis, miR-429 expression levels were divided into two groups: (1) low expression, 2^−ΔCt^ ≤ 2; and (2) high expression, 2^−ΔCt^ > 2, according to the average value among HCC cases. For the relative expression of miR-429 in cancer cells, miR-429 expression was normalized to endogenous controls U6 by the comparative CT method (2^−ΔΔCt^ method [24]).

### 2.4. Cell Lines and Culture

The QSG-7701 cells (a kind of peritumoral liver cells) and SMMC-7721 cells (a kind of HCC cancer cells) were obtained from the Cell Bank of Shanghai Institute of Cell Biology of the Chinese Academy of Sciences. Cells were cultured in DMEM medium (HyClone, Thermo Fisher Scientific (China) CO., Ltd., Shanghai, China) containing high glucose and L-glutamine supplemented with 10% fetal bovine serum at 37°C in an atmosphere of 5% CO_2_/100% humidity. All experimental analyses were done with cells in logarithmic growth. Cells were determined to be free of Mycoplasma.

### 2.5. Cell Transfection and Cell Proliferation and Apoptosis Assay

Cells were transfected with an miR-429 mimic, its inhibitor, its mock, or null control (GenePharma, China) using Lipofectamine 2000 (cat# 11668-027, Invitrogen Grand Island, NY, USA) according to the manufacturer's instructions. The cell proliferation assay was done with the 3-(4,5-dimethylthiazol-2-yl)-2,5-diphenyltetrazolium bromide (MTT) reduction assays. Cells were seeded into 96-wells plates for MTT analysis. Twenty-four hours after cell transfections, 20 *μ*L of MTT (5 mg/mL) was added into each well and incubated for 4 hours. After that, the supernatant was discarded, and then 150 *μ*L dimethyl sulfoxide was added to each well and oscillated for 10 min to dissolve the precipitate. Finally, OD absorbance (at 490 nm) was measured using UV spectrophotometer at 24 hours, 48 hours, and 72 hours after transfection. The assay was performed three times in eight replicates. 

In this study, we used flow cytometry technique to elucidate cell apoptosis. Cells were seeded in 6 wells, and the transfections were performed when cells reached 70% confluent. Forty-eight hours after transfection, cells were harvested, washed, and resuspended for cell counting analysis and apoptosis.

### 2.6. AFB1 Toxicity Analysis

AFB1 toxicity value was evaluated as our previous methods [19]. Briefly, 48 hours after transfections, cells were treated with AFB1 (Sigma) at final concentrations of 24 nM for 1 day, and then the DNA was extracted for AFB1-DNA adduct analysis.

### 2.7. Statistical Analysis

All analyses were performed with the statistical package for social science (SPSS) version 18 (SPSS Institute, Chicago, IL, USA). MiR-429 expression among different tissues and cells was compared by independent two-sample *t*-test for two groups or one-way ANOVA with Bonferroni corrections for three or more than three groups. Nonconditional logistic regression was used to evaluate odds ratios (ORs) and 95% confidence intervals (CIs) for the effects of miR-429 expression on the pathological features of HCC. Kaplan-Meier survival analysis with the log-rank test was used to evaluate the relationship between miR-429 expression and HCC prognosis. Hazard ratios (HRs) and 95% CIs for miR-429 expression were calculated from multivariate Cox regression model. In this study, a *P* value of <0.05 was considered statistically significant.

## 3. Results

### 3.1. The Characteristics of HCC Patients

The demographic data of the patients are shown in Table 1. The present study comprised of 138 HCC patients with 125 (90.6%) males and 13 (9.4%) females. The mean age was 46.7 ± 11.7 years. The HBV and HCV infective rates were 84.1% (116 of 138) and 1.4% (2 of 138), respectively. One hundred percent cases featured HCC with I-II TNM stage and received the same curative resection treatment. Most of them had liver cirrhosis and featured AFB1 exposure. In this study, we elucidated AFB1 exposure levels through testing AFB1-DNA adducts of DNA samples from cancerous tissue of the patients and found the mean of 2.98 ± 1.48 *μ*mol/mol DNA. This suggested that higher levels of adducts were in DNA samples from cancer tissues than in those from peripheral blood [20].

### 3.2. MiR-429 Was Downregulated in HCC Samples and in SMMC-7721 Cells

**Figure 1:**
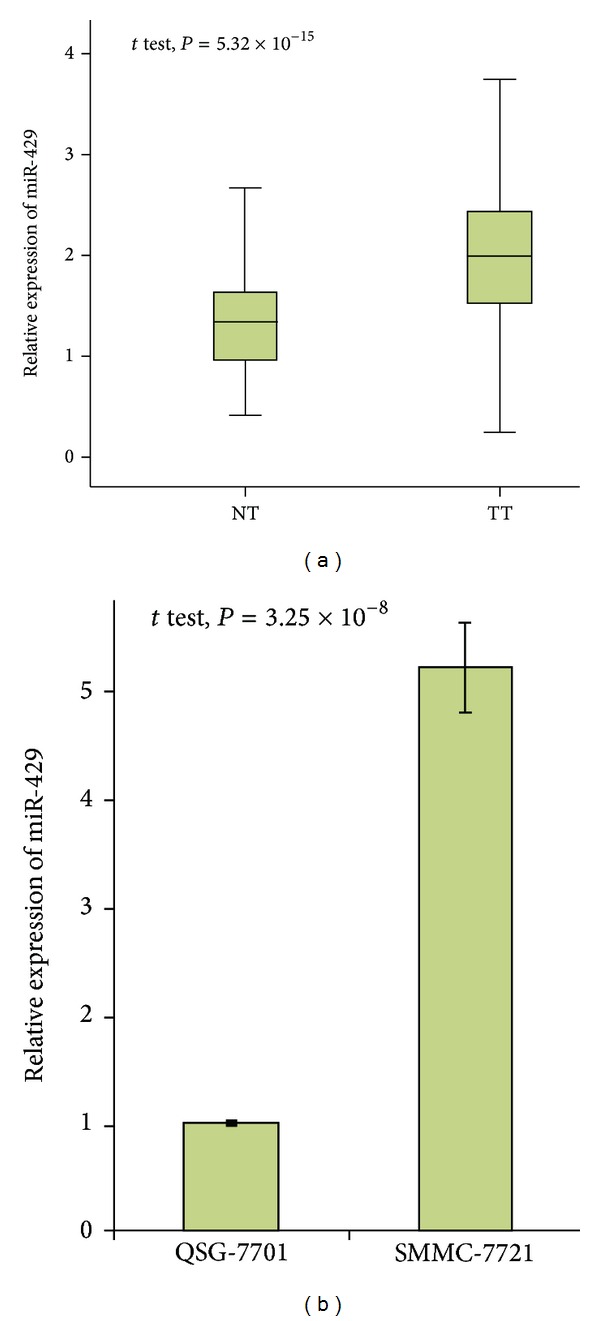
MiR-429 expression correlated with HCC tumorigenesis. (a) MiR-429 expression was evaluated in the tumor tissues, compared with in the adjacent noncancerous tissues. The relative expression of miR-429 is shown as box plots, with horizontal lines representing the median, the bottom, and the top of the boxes representing the 25th and 75th percentiles, respectively, and vertical bars representing the range of data. We compared the difference among groups using the Student *t*-test. (b) MiR-429 expression was higher in cancer cell line SMMC-7721 than in noncancer cell line QSG-7701.

We analyzed the expression of mature miR-429 and U6 RNA in HCC tumor tissues and adjacent noncancerous tissues and evaluated the significance of differential miR-429 expression by comparing Ct values in these two types of tissues. We observed that 132 patients had significantly higher levels of miR-429 expression in tumour tissues than in nonmalignant adjacent liver tissues. The average expression of miR-429 was significantly higher in HCC tumor samples (TT), when compared with adjacent noncancerous tissues (NT, Figure 1(a)). We also found similar results in the expression analysis of this microRNA in HCC cell lines SMMC-7721 and nontumor liver cell lines QSG-7701 (Figure 1(b)).

### 3.3. MiR-429 Expression Associated with Tumor Sized and AFB1-DNA Adduct Levels

To investigate the association between miR-429 expression and clinicopathological features of HCC, we divided miR-429 expression in cancerous tissues into two groups: low expression group (relative level ≤ 2) and high expression group (relative level > 2), according to the average expression levels. We next analyzed the distribution difference of this microRNA expression among different clinicopathological characteristics of cases. Results showed that miR-429 expression levels modified tumor size (OR = 3.13, 95% CI = 1.47–6.67) and AFB1-DNA adduct quantity (OR, 2.70; 95% CI, 1.28–5.56, Table 2) but did not affect other features (data not shown).

### 3.4. MiR-429 Expression Correlated with HCC Prognosis

**Figure 2:**
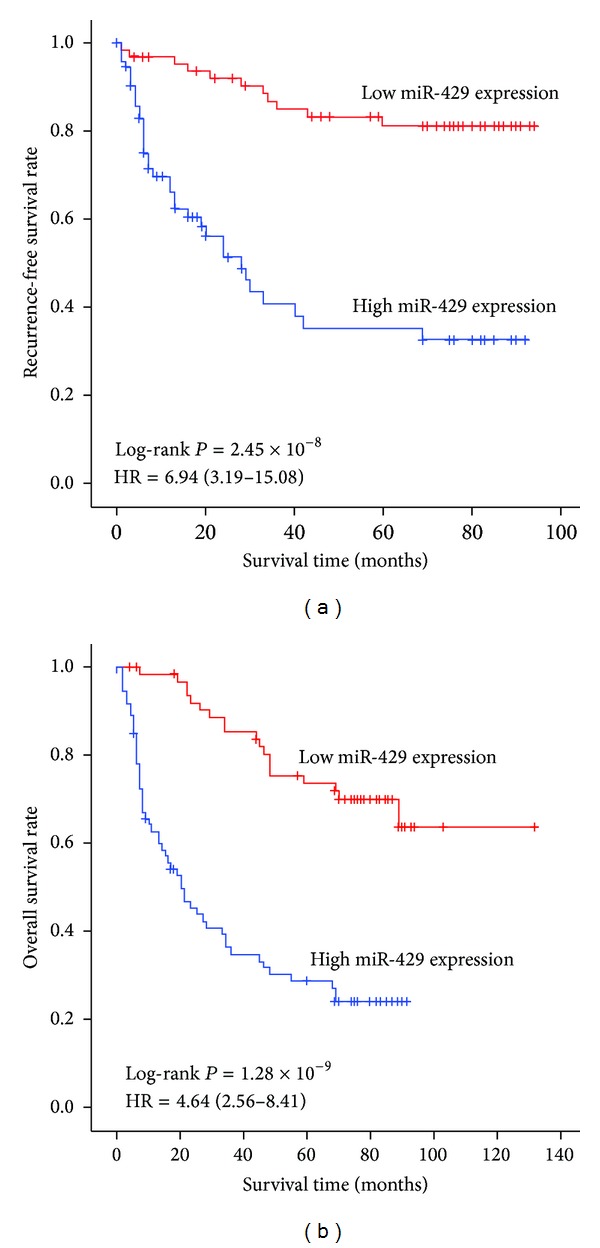
Association between survival and miR-429 expression in 138 HCC cases receiving curative treatment. According to the average expression in cancerous tissues, miR-429 expression was divided into two groups: low expression group (relative level ≤ 2) and high expression group (relative level > 2). MiR-429 expression was associated with tumor reoccurrence-free survival (a) and overall survival (b) of HCC. Cumulative hazard function was plotted by Kaplan-Meier's methodology, and *P* value was calculated with two-sided log-rank tests. Relative hazard ratio (HR) and corresponding 95% CI of high miR-429 expression (compared with low expression) were calculated using multivariable cox regression model (including all significant variables).

**Figure 3:**
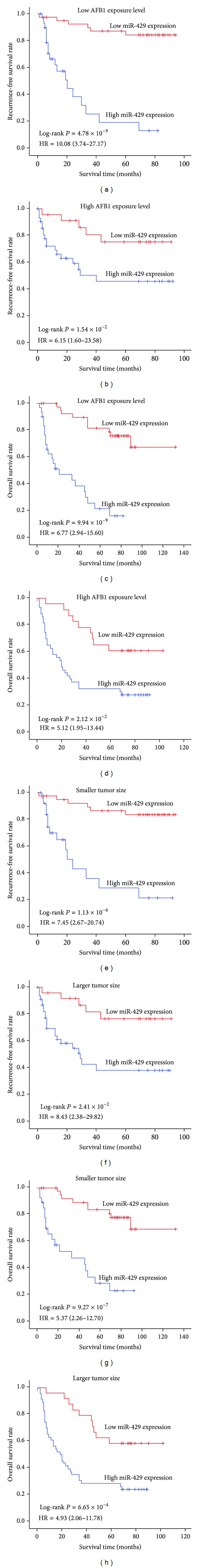
Survival analysis of miR-429 expression in strata of AFB1-DNA adducts and tumor size. ((a)-(b)) Tumor recurrence-free survival (RFS) and miR-429 expression in strata of AFB1-DNA adduct levels. ((c)-(d)) Overall survival (OS) and miR-429 expression in strata of AFB1-DNA adduct levels. ((e)-(f)) RFS and miR-429 expression in strata of tumor size. ((g)-(h)) OS and miR-429 expression in strata of tumor size. Cumulative hazard function was plotted by Kaplan-Meier's methodology, and *P* value was calculated with two-sided log-rank tests. Relative hazard ratio (HR) and corresponding 95% CI of high miR-429 expression (compared with low expression) were calculated using multivariable cox regression model (including all significant variables).

During the follow-up period of 138 HCC patients, 47 faced tumor recurrence with 60.1% of the 5-year RFS rate, and 72 died with 62.3% of the five-year OS rate. Kaplan-Meier survival analysis exhibited that patients with high miR-429 expression featured a significantly poorer prognosis than those with low miR-429 expression (*P* = 2.45 × 10^−8^ for RFS and *P* = 1.28 × 10^−9^ for OS, respectively, Figure 2). Multivariate cox regression analysis (with stepwise forward selection based on likelihood ratio test) was performed to determine whether miR-429 expression was an independent predictor of RFS for patients with HCC. The results showed that miR-429 expression was significantly associated with poorer RFS prognosis (HR = 6.94, 95% CI = 3.19–15.08, *P* = 9.94 × 10^−7^, Figure 2(a)). Risk role was also found in the OS analysis; the corresponding HR (95% CI) for high miR-429 expression was 4.64 (2.56–8.41), with a *P* value 4.11 × 10^−7^ (Figure 2(b)). Next stratified analysis showed similar risk value (Figure 3). Taken together, these results showed that this microRNA is independent of other clinical covariates and suggested that it could be used as an independent prognostic factor for HCC.

### 3.5. MiR-429 Expression Modified SMMC-7721 Cell Proliferation

**Figure 4:**
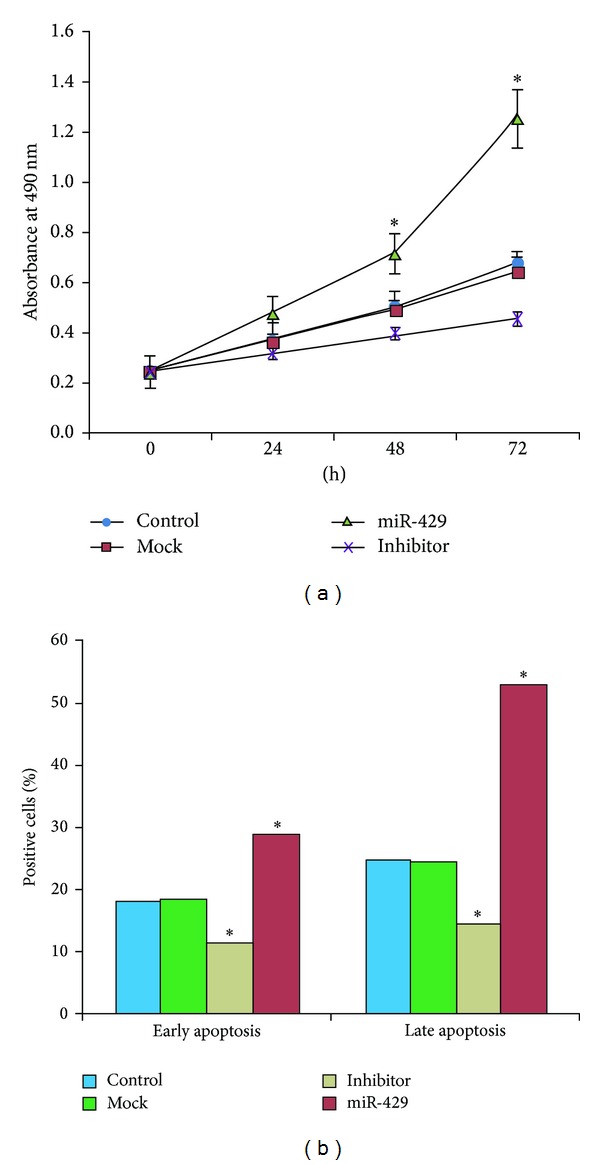
MiR-429 expression modified the proliferation and the apoptosis of HCC cancer cells. According to the types of mimics transfected, cell lines were divided into four groups: control group (Control, by null mimics), mock group (Mock, by mock mimics) miR-429 group (miR-429, by mature miR-429 mimics), and inhibitor group (Inhibitor, by inhibitor of mature miR-429). (a) Association between miR-429 expression and cancer cell proliferation was elucidated using the 3-(4,5-dimethylthiazol-2-yl)-2,5-diphenyltetrazolium bromide (MTT) reduction assays. (b) Relationship between miR-429 expression and cancer cell apoptosis was evaluated by flow cytometry technique. Data were analyzed using one-way ANOVA with Bonferroni corrections. *P < 0.05.

We evaluated the functional role of miR-429 in liver cancer cells by means of measuring cell proliferation in SMMC-7721 cells which were transfected with miR-429 mimic and its inhibitor. In this study, according to the types of mimics transfected, cell lines were divided into four groups: control group (Control, by null mimics), mock group (Mock, by mock mimics), miR-429 group (miR-429, by mature miR-429 mimics), and inhibitor group (Inhibitor, by inhibitor of mature miR-429). MTT assays were next employed to detect the proliferation of SMMC-7721 cell lines. Overexpression of miR-429 in SMMC-7721 cells promoted proliferation, while downregulation of miR-429 in SMMC-7721 cells inhibited cell proliferation. The proliferation of tumor cells in the miR-429 groups, compared with the control group, noticeably increased at 48 h and 72 h (*P* < 0.05, Figure 4(a)). On the other hand, compared with the mock group, the proliferation of tumor cells in the inhibitor groups was inhibited significantly at 48 h and 72 h (*P* < 0.05, Figure 4(a)).

### 3.6. MiR-429 Expression Modulated SMMC-7721 Cell Apoptosis

We also investigated the function of miR-429 in SMMC-7721 cells through analyzing changes in apoptosis after the liver cancer cells were transfected with miR-429 mimic and its inhibitor. DNA content of transiently microRNA-transfected cells was analyzed by flow cytometry. The early and late apoptosis of SMMC-7721 cell lines in the miR-429 group was significantly inhibited (*P* < 0.05) compared with the control group. Tumor cell apoptosis in the inhibitor group, compared with the mock group, was promoted significantly (*P* < 0.05) (Figure 4(b)).

### 3.7. MiR-429 Expression Increased AFB1-DNA Adducts in the SMMC-7721 Cells

**Figure 5:**
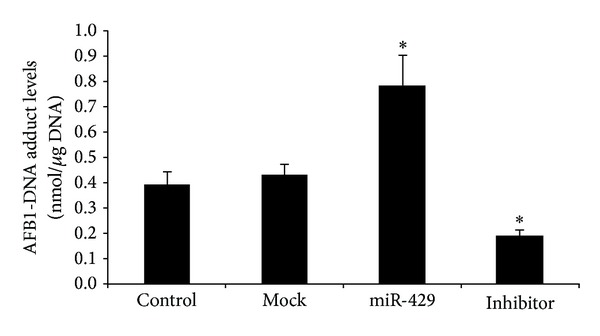
AFB1-DNA adducts formation in AFB1-treated SMMC-7721 cells with overexpression of miR-429 (see Section 2). Levels of AFB1-DNA adducts were tested using comparative ELISA. Data were analyzed from three independent tests using one-way ANOVA with Bonferroni corrections. *P < 0.05.

To explore the effects of miR-429 expression on AFB1-DNA formation, we accomplished a toxin experiment of AFB1 in the SMMC-7721 cells transfected by different mimics. Results showed that group with overexpression of miR-429 had elevated levels of AFB1-DNA adducts (0.78 ± 0.12 nmol/*μ*g DNA) compared with control group (0.39 ± 0.05 nmol/*μ*g DNA, *P* < 0.05, Figure 5). On the other hand, compared with mock group (0.43 ± 0.04 nmol/*μ*g DNA), cells transfected by miR-429 inhibitor featured decreased levels of DNA adducts (0.18 ± 0.02 nmol/*μ*g DNA, *P* < 0.05, Figure 5).

## 4. Discussion

HCC is the most common histological type of liver cancer in the world [3, 7]. More than 80% of all HCC cases occur in developing countries, and approximately 55% of all cases occur in China (especially in the southeast areas such as Guangxi Zhuang Autonomous Region) [3]. Because of metastasis or other causes, most HCC cases are already in an incurable stage with an extremely poor prognosis at the time of diagnosis [6, 25]. Therefore, new prognosis biomarkers and therapies have been expected, but no remarkable advances have been made in the treatment and prognostic prediction of this malignant tumor [4].

Increasing number of studies has shown that microRNAs may be a type of significant prognosis factor and potential therapeutic target. MicroRNAs are a class of small noncoding single-stranded RNA and typically contain 18–24 nucleotide sequence [26–30]. Originally, they were found as an evolutionarily conserved class of small RNAs which are formed from the sequential processing of primary transcripts by two RNase enzymes, Drosha and Dicer [28–30]. To date, it has been identified that there are more than 1,800 microRNAs in the mammalian genome (miRDatabase). Functionally, microRNAs are involved in regulating gene expression and play a role in a very wide range of physiological processes including cell differentiation, cell proliferation, cell apoptosis, physiological timing, metabolism, and hormone secretion [31–33]. Furthermore, increasing evidence has exhibited that this type of small RNAs may play a role in the aetiology and pathogenesis of various cancers by targeting a number of oncogenes or tumour suppressors genes [34]. Recent several reports have shown that the dysregulation of some microRNAs expression may modify the prognosis and the clinicopathological features of tumors such as colon cancer [35, 36], gastric cancer [37, 38], lung cancer [39], and skin cancer [34, 40]. 

Of particular recent interest is the possible contribution of miR-429 to tumor prognosis and clinicopathological characteristics [13, 15, 17, 18, 41, 42]. MiR-429 is classified as a member of miR-200 family and may play an important role in tumor prognosis. For example, Li et al. [15] investigated the correlation between miR-429 expression and colorectal cancer. They found that miR-429 expression is higher in the cancerous tissues than nontumor tissues. Survival analysis showed that the expression of this microRNA modified colorectal cancer outcome and was an independent prognostic factor for malignant tumor. In this study, we collected 138 HCC tissue samples from Guangxi Zhuang Autonomous Region, a high epidemic area of HCC in China, and explored the possible effects of miR-429 expression on HCC prognosis using TaqMan-PCR technique. We found that these patients having high miR-429 expression in the tumor tissues had a significant poor RFS and OS after curative resection compared with those with low expression of miR-429. Multivariate cox regression analysis exhibited high expression of this microRNA that increased about 6-times tumor reoccurrence risk and 4-times death risk; moreover, this risk did not depend on the clinicopathological change. Taken together, these data suggested that miR-429 expression should be an independent prognostic factor for HCC and that its aberrant expression could be used as a prognostic marker for this tumor. Therefore, it is well known that postoperative adjuvant therapy might significantly improve HCC prognosis.

Recently, Li et al. [15], Sun et al. [13], Liu et al. [17], Hashimoto et al. [16], and Chen et al. [18] have shown that miR-429 expression may modulate the tumorigenesis of gastric cancer and ovarian cancer. To explore whether this microRNA modified liver tumorigenesis, we tested expression difference of miR-429 in different tissue samples and cells and analyzed the effects of this microRNA on cell function in vitro. Our results showed that miR-429 was markedly upregulated in human HCC tumor tissues compared with adjacent noncancerous tissues. This different expression was also confirmed in cell culture in vitro. In the next analysis of cellular function, we only investigated the effects of miR-429 on liver cell proliferation and apoptosis, mainly because of the following reasons. On the one hand, infinite proliferation capacity is a key characteristic of malignant tumors [7]. On the other hand, apoptosis is a major barrier that must be circumvented during tumor development, and evasion of apoptosis is considered to be one of the major hallmarks of tumorigenesis [43]. Reduced rates of apoptosis within the liver tissues are associated with increased risk of HCC [7, 44]. Our results exhibited that the overexpression of miR-429 progressed cell proliferation and inhibited cell apoptosis. On the contrary, the suppression of miR-429 expression hindered cell proliferation and promoted cell apoptosis. These data suggest that this microRNA plays an important role in liver tumorigenesis, and functionally acts as an oncogene in HCC. Supporting our hypothesis that miR-429 has an oncogenic role, recent several studies have exhibited that it can promote the carcinogenesis of pancreatic ductal adenocarcinoma, gastric cancer, and colorectal cancer by targeting EP-300, SOX2, or c-myc [15, 16, 42], while the inhibitors of miR-429 significantly suppressed such tumor cells as of endometrial cancer cells and gastric cancer cell proliferation. However, this microRNA is shown to have a tumor-suppressor function in breast cancer and gastric cancer [13, 17]. The different function of miR-429 in different types of cancer may be because of the differences of cellular context or alternatively the targeted genes.

In the present study, we explored the relationship between miR-429 expression and the clinicopathological features of HCC and found this microRNA only modulated tumor size. These patients with high miR-429 expression would face higher tumor size, suggesting that this microRNA might promote tumor growth and subsequently might play an important role in the carcinogenesis of HCC.

Additionally, we also investigated the association between miR-429 expression and AFB1, mainly because AFB1 is a major carcinogen for liver cancer in China, especially Guangxi Zhuang Autonomous Region [1]. This carcinogen is produced by fungi of the *Aspergillus spp*. and metabolized mainly by cytochrome P450 into the genotoxic metabolic AFB1-exo-8,9-epoxide (AFB1-epoxide). AFB1 epoxide is able to bind to DNA and causes the formation of AFB1-DNA adducts. Increasing evidence has shown that the levels of AFB1-DNA adducts correlate with HCC risk and prognosis, whereas the formation process of AFB1-DNA adducts can be modified by some factors such as detoxifying enzymes and DNA repair enzymes [1, 2, 45], while our present studies exhibited that miR-429 expression promoted AFB1-DNA adducts formation and increased adducts mount in liver cancer tissues. The aforementioned results were furthermore proved in the toxicological analysis of AFB1 in vitro. This is possibly because miR-429 can target some detoxification enzyme genes and/or DNA repair genes and reduce their detoxification capacity or DNA repair capacity and subsequently increase DNA damage and promote AFB1-DNA adducts formation. These results provided new insights into the mechanism of HCC induced by AFB1.

The present study had several limitations. Only 138 HCC patients were enrolled in the analysis of the clinicopathological characteristics and prognosis. We would like to confirm the findings in a larger liver cancer patient population. Another important limitation is that we did not do migration and invasiveness assays to validate the involvement of miR-429 in tumour migration and invasion.

## 5. Conclusions

In summary, this study is, to the best of our knowledge, the first report that describes miR-429 expression in liver cancer and its associations with HCC prognosis. Our results showed that miR-429, as an oncogene, was overexpressed in liver cancer tissues and could be considered as a potential prognostic factor for HCC. Furthermore, overexpression of this microRNA promoted proliferation and inhibited apoptosis in liver cancer cells. Therefore, more detailed molecular pathogenesis analysis deserves elucidation based on the results from large samples. Expanding insights into the key role of dysregulated microRNAs involved in liver tumorigenesis will yield important clues for the complicated molecular pathogenesis of HCC and may assist the development of new therapeutic regimens for HCC patients.

## Figures and Tables

**Table 1:** Characteristics of the patients with HCC.

Characteristics	
Age, yr	
Mean ± SE	46.7 ± 11.7
Range	15–75
Sex	
Man, *n* (%)	125 (90.6)
Female, *n* (%)	13 (9.4)
Ethnicity	
Han, *n* (%)	107 (77.5)
Zhuang, *n* (%)	31 (22.5)
HBV infection	
HBsAg (−), *n* (%)	22 (15.9)
HBsAg (+), *n* (%)	116 (84.1)
HCV infection	
anti-HCV (−), *n* (%)	136 (98.6)
anti-HCV (+), *n* (%)	2 (1.4)
Smoking status	
No, *n* (%)	109 (79.0)
Yes, *n* (%)	29 (21.0)
Drinking status	
No, *n* (%)	110 (79.7)
Yes, *n* (%)	28 (20.3)
AFB1 exposure	
low, *n* (%)	72 (52.2)
High, *n* (%)	66 (47.8)
Liver cirrhosis	
No, *n* (%)	2 (1.4)
Yes, *n* (%)	136 (98.6)
TNM stage	
I, *n* (%)	8 (5.8)
II, *n* (%)	130 (94.2)
Tumor size	
≤5 cm, *n* (%)	68 (49.3)
>5 cm, *n* (%)	70 (50.7)
Total, *n* (%)	138 (100)

**Table 2:** Expression levels of miR-429 and AFB1 exposure levels and tumor size of cases.

	Low expression		High expression		OR (95% CI)^a^	*P*
	*n*	%	*n*	%		
AFB1 exposure						8.88 × 10^−3^
Low level	42	64.6	30	41.1	Reference	
High level	23	35.4	43	58.9	2.70 (1.28–5.56)	
Tumor size						3.10 × 10^−3^
≤5 cm	41	63.1	27	37.0	Reference	
>5 cm	24	36.9	46	63.0	3.13 (1.47–6.67)	

^a^Adjusted by age, sex, race, HBV and HCV infection status, and smoking and drinking status.
